# Insights into fundamental problems of rockburst under the modern structure stress field

**DOI:** 10.1038/s41598-022-24857-4

**Published:** 2022-11-24

**Authors:** Hai Rong, Nannan Li, Hongwei Zhang, Dequan Sun, Bingjie Huo

**Affiliations:** 1 College of Mining, Liaoning Technical University, Fuxin, 123000 China; 2 Engineering Laboratory of Deep Mine Rockburst Disaster Assessment, Jinan, 250104 China; 3 Shandong Province Research Institute of Coal Geology Planning and Exploration, Jinan, 250104 China

**Keywords:** Natural hazards, Environmental impact, Structural geology, Geodynamics

## Abstract

Rockbursts are some of the most severe dynamic disasters in coal mines. In this paper, the discrimination method of the tectonic stress field is proposed by analyzing the modern stress field in China. The tectonic stress field formed by modern tectonic movement guides in situ stress measurements. According to the stress state classification, most rockbursts in coal mines in China are closely associated with tectonic stress. For tectonic stress-driven rockbursts, modern tectonic movement and modern tectonic stress fields must be considered. The stress change and energy transfer caused by tectonic movement affect the geological structure where coal mines are located. Energy accumulation under rockburst conditions is mainly formed by natural geo-dynamic movement and the mining configuration, and energy accumulation is the basis for rockbursts. The application of the geo-dynamic environmental evaluation method to determine the coalfield geo-dynamic process and the influence of modern tectonic movement is proposed. Accordingly, the classification method of rockbursts in coal mines is established. Based on the distribution characteristics of modern tectonic conditions in China, it is revealed that these dynamic disasters follow a “110” distribution. Finally, a “three condition” criteria of rockbursts is proposed: the geo-dynamic environment is a necessary condition for rockbursts, mining disturbance is a sufficient condition for rockbursts, and risk-releasing measures are a condition controlling rockburst risk mitigation.

## Introduction

In 1738, the first documented rockburst occurred in the Leipzig coal mine in the South Stafford coalfield in Great Britain. Afterward, rockbursts were reported in dozens of countries and regions, including the former Soviet Union, South Africa, Germany, America, Canada, India, Britain and China^1^. China is the world’s largest coal producer and consumer. Due to the increasing energy demand for rapid economic development and the scarcity of shallow coal resources, deep underground coal mining is dominating. As the mining depth increases and the mining intensity becomes stronger, rockburst-related disasters are correspondingly more severe. In the last fifty years, rockburst-related, dynamic pressure-induced disasters have been an intense academic topic for rock mechanics researchers in China due to their complexity and serious consequences.

Beginning in the 1950s, the theories used to explain the cause and mechanism of rockbursts include strength theory, stiffness theory, energy theory, burst-prone theory and instability theory. In 1975, Brauner^2^ proposed coal clamping theory. In this theory, the hard roof tightly clamps the coal, which hinders unloading deformation of the coal itself and the coal-surrounding rock system. As the lateral pressure prevents unloading movement of the coal, the coal becomes tighter, sustains a higher pressure and thus stores higher elastic energy. Once the pressure is suddenly raised or the system resistance decreases, the coal is broken, moves and is ejected to the mining space, which forms the dynamic pressure.

In the 1960s, Cook^3^ proposed the stiffness theory in which a rockburst occurs when the stiffness of the surrounding rock support is less than the mining system stiffness. The fundamental reason for rockburst occurrence is that the mining system stiffness reaches the limit stiffness.

Avieshin^4^ in the 1950s and Cook et al.^5^ in the late 1960s proposed the energy theory, i.e., when the released energy of the support-surrounding rock structure exceeds its dissipated energy, a rockburst occurs. The rockburst energy equals the difference between the released energy and the dissipated energy.

Petukhov^6^ stated that the elastic energy of the surrounding rock and the energy stored in the coal provide the energy base for rockbursts. Zhao^7^ used the minimum energy principle to explain the elastic deformation and plastic damage of coal and rock and suggested that the energy released upon rock failure is substantially higher than the energy required for the rock to fail. Zhao^8^ investigated the energy dissipation of coal and rocks preceding a rockburst occurrence based on the dissipated structure theory and equilibrium thermodynamics and proposed a corresponding rockburst instability evaluation method.

Based on the relationship between rockbursts and rock instability, Zhang^9^ proposed the coal and rock mass deformation and instability theory. The principle of this theory is that rockbursts stem from coal and rock mass instability and failure caused by external forces. The tremendous energy released by instability leads to the occurrence of rockbursts.

Xie^10^ applied fractal theory to elucidate the mechanism and characteristics of rockbursts and reckoned that rockburst occurrence is the process of microfractures developing into macrofractures via damage. The fractal dimension is negatively proportional to the degree of microfracture development. A minimum fractal dimension indicates the highest possibility of a rockburst.

Qi^11^ proposed the ‘three factors’ theory of rockbursts, including the rockburst-prone factor, structure factor and stress factor. The stress factor or condition is the core requirement for a rockburst. Most rockburst incidents occur in tectonic regions, such as faults. Rockburst occurrence is intimately associated with rockburst proneness, which is also closely related to high-stress and high-energy environments.

Pan^12^ considered coal compression, roof breakage, and fault slippage as the three factors inducing rockbursts. He classified rockbursts into three types, namely, the coal compression type, roof breakage type and fault slippage type.

Dou^13^ developed the “mitigating bursts by reducing the strength” model. The principle of the model is to reduce the burst proneness by deconstructing the energy accumulation of the coal and rock mass, which can be achieved by a loosened coal and rock mass.

Jiang^14^ stated that a rockburst is caused by the stable to unstable transition of the coal to a rock mass and the progressive development of internal fissures to macroscopic failure via multiple inducing factors under various geological and mining conditions. Rockbursts are induced by a combination of multiple factors, such as the geological environment, mining disturbance degree, and stress and energy field evolution.

Focusing on the invisibility, spontaneity, and lag of the ‘creep type’ rockburst, Jiang^15^ established the three-dimensional creep model and corresponding creep equations. The evolution of creep relies on a superthick coal seam and high in situ stress. The coal and rock mass strength and resistance are reduced during creep, which finally generates a creep-type rockburst in the weak region of the roadway.

Pan^16^ theoretically analyzed the mechanism of rockbursts occurring in the floor of thick coal seams in tectonic regions and wall–roof-type rockbursts. He suggested that floor-type rockbursts originate from stress concentration and result in the deformation of the bottom coal, and substantial energy is stored in the floor. When the horizontal stress exceeds the limit, the sudden energy release leads to rockburst occurrence. Wall–roof-type rockbursts are developed because over mining operations, the roadway wall fails, which decreases the friction between the roof and the roadway wall, unbalances the stress distribution and thus squeezes out the coal toward the roadway.

Tan^17^ investigated the mechanical mechanism of rockbursts and suggested that the release of the elastic energy stored in the roof, floor and coal leads to the dynamic failure of the roadway wall in deep underground coal mining, which is the basic mechanism of rockbursts.

Pan^18^ proposed the rockburst initiation theory and suggested that rockbursts result from dynamic instability of the combined structure, formulated by the failure of an elastic–brittle single structured material after its strength is exceeded. He also studied the static and dynamic sources in deep underground coal mines and classified three rockburst types, i.e., static and dynamic combination type, high static loading type, and high static unloading type^19^.

According to the loading state and failure characteristics of coal and rock masses, Tai^20^ classified rockbursts into three types, namely, compression of the coal and rock mass type due to excavation disturbance, tension of the roof and floor type and shear slip of the fault type.

Zhu^21^ grouped the rockburst types due to integral instability, including integral instability of the isolated working face, the main roadway coal pillar, and the bottom coal slip.

From the viewpoint of the carrier of energy storage and release, Shi^22^ divided rockbursts into three types, namely, the energy storage and release of the coal, roof, and fault zone and surrounding rock.

Zhang^23,24^ proposed that the dynamic coal mine disasters of different coalfields, different coal mines and different tectonic and stress conditions vary from site to site. Han^25^ proposed the tectonic sag concept and quantitative evaluation index and revealed that the tectonic contrast controls dynamic coal mine disasters. Song^26^ examined the regional tectonic and stress conditions of coal mines and established the governing of tectonic stress zones based on dynamic coal mine disasters. Zhang and Li^27^ developed the multifactor probability prediction method for discriminating dynamic coal mine disasters; this method quantifies the dynamic disaster risk of each zone. Zhang and Lan^28^ built a geo-dynamic environmental evaluation approach for dynamic coal mine disasters and disclosed the dynamic background of the dynamic disasters. Rong^29^ proposed a computing method for the dynamic coal and rock system at the structural scale and established the risk degree of coal and rockbursts at different scales. This paper discusses the fundamental issues of rockbursts based on the analysis of the modern tectonic stress field.

## Introduction to rockbursts

Rockburst refers to the dynamic phenomenon of sudden and violent failure caused by instantaneous release of the elastic energy accumulated in the coal and rock around the roadway or working face in coal mines. It is often accompanied by instantaneous movement, ejection, loud noise and air blasting. In China, coal mine rockbursts are characterized by outbursts and complexity, and they are an evolving damage and catastrophic process induced by the progressive fracturing of complex engineering geological media with strong heterogeneity, discontinuity and nonlinearity under multifield coupling action. The nature of rockbursts is the initiation, development, coalescence and final instability of microfractures induced by mining disturbance or increased strain.

**Figure 1:**
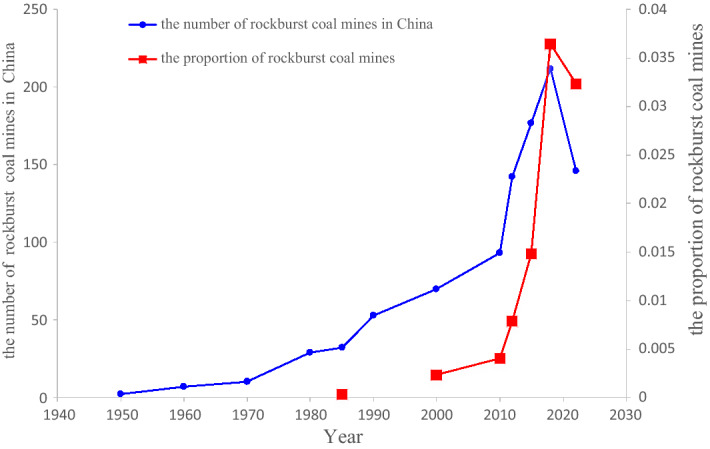
The number of rockburst coal mines in China.

**Figure 2:**
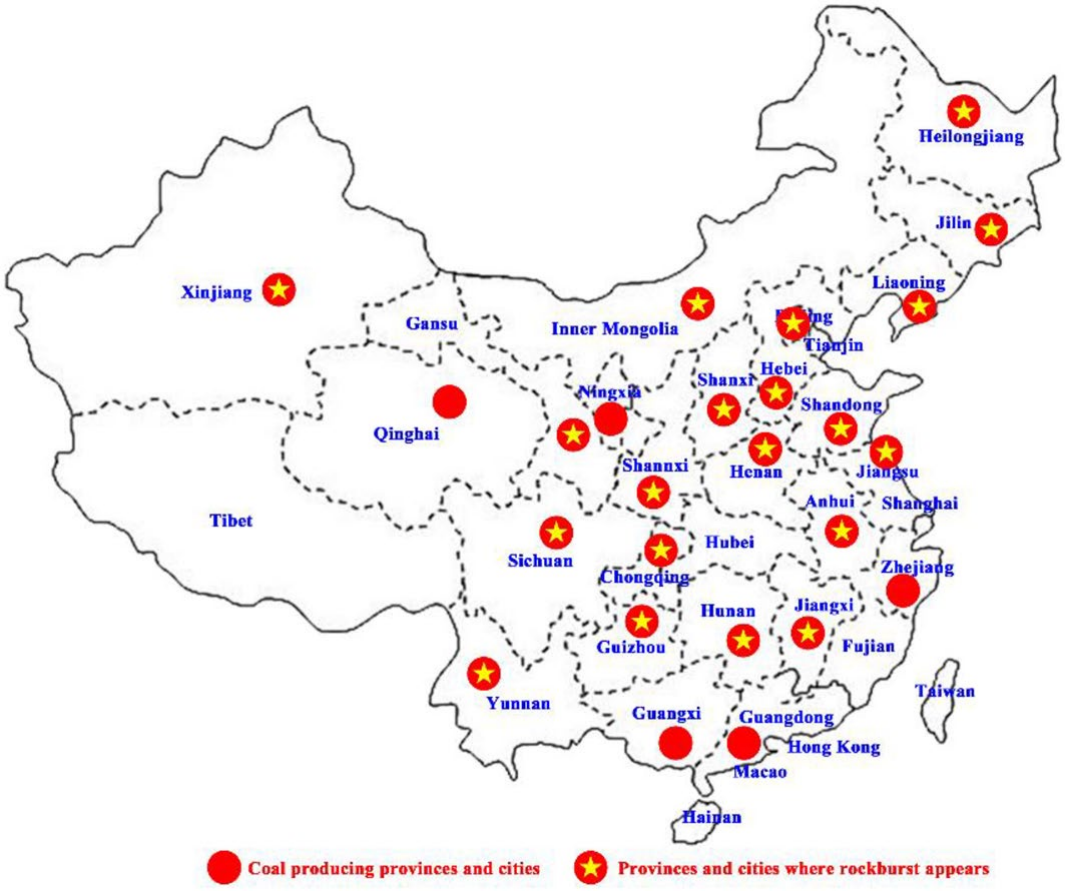
Rockburst coal mine distribution in China.

The rock strength increases and its capacity for storing elastic energy is correspondingly improved as the mining progresses to deeper depths. In the deep underground mining environment, the existence of high in situ stress and strong mining disturbance promotes the release of tremendous energy stored in the rock. Variations in stress, mining and energy conditions further aggravate the risk of rockbursts. The first documented rockburst incident in China occurred in the Shengli coal mine of the Fushun Mining area in 1933. With the increase in the coal mining scale and coal production, the number of rockburst coal mines is also increasing annually. In 1985, more than 32 rockburst coal mines occurred among the 80,000 total Chinese coal mines, and the proportion of rockburst coal mines was 0.04%. In 2018, 212 rockburst mines occurred in more than 5800 coal mines across China, and the proportion of rockburst coal mines was 3.7%. In 2022, 146 rockburst mines occurred in more than 4500 coal mines across China. Even though the number of rockburst coal mines decreases based on the overall number of coal mines in China, the proportion of rockburst coal mines can still reach 3.2%. The number of rockburst coal mines in China is shown in Fig. 1. Twenty provinces (cities and autonomous regions) have recorded rockburst incidents (Fig. 2). The surge in the number of rockburst coal mines in China after 2000 is mainly due to the continuous growth in both coal mining intensity and extraction depth. Rockbursts have become one of the main geological disasters in Chinese coal mines. According to incomplete statistics, 13 rockburst incidents have occurred in China in the past 10 years, causing nearly 100 deaths and nearly 1000 injuries. As shallow coal resources become scarce, the number of coal mines deep underground is growing yearly, and dynamic disasters such as rockbursts increasingly threaten the safety of coal mines.

## Rockburst classification and stress field discrimination

### Rockburst classification

The current rockburst classification method is based on the stress state of the coal and rock mass, the exhibited burst strength, the seismicity magnitude, the amount of ejected coal, and the locations of occurrence. In this paper, rockburst types according to the stress state of the coal and rock mass are classified. The classification method is shown as follows^30,31^.

1. Gravity-driven rockburst: the rockburst is caused mainly by gravity, with no or only negligible tectonic stress.
2. Tectonic stress-driven rockburst: the rockburst is mainly caused by tectonic stress, which is remarkably higher than gravity.
3. Gravity and tectonic stress-driven rockburst: the rockburst is mainly caused by the combination of gravity and tectonic stress.

Is this classification reasonable, and is it consistent with the actual stress state of the coal mine? Since rockbursts are classified according to the stress state of the coal and rock mass, the stress field type where the coal mine is located must first be determined. The geostress field is usually divided into the gravity stress field and tectonic stress field. The gravity stress field is caused by the weight of the overlying strata, and the tectonic stress field is the stress state of various tectonic traces that have a genetic relation in a certain area. Both fields are components of the natural stress field. The gravity stress field generated by the self-weighting of the overlying strata is distributed uniformly in space. The tectonic stress field produced by tectonic movement can be categorized into the local tectonic stress field, regional tectonic stress field and global tectonic stress field, depending on the scale. Based on time, it can be grouped into a modern tectonic stress field and a paleotectonic stress field, and the modern tectonic stress field can be estimated only by in situ stress measurements. The tectonic stress field is a function of space and time and varies with the development of tectonic traces (Supplementary Information).

### Method for determining the stress field type

Determination of the coal mine stress field type is the key to rockburst classification based on the stress state of the coal and rock mass. According to the classical Zernike hypothesis (Eq. 1), the stress field following Eq. (1) is the gravity stress field; otherwise, it is the tectonic stress field. The key parameter is the lateral pressure coefficient $\lambda$, as well as the Poisson’s ratio $\mu$.

1 $$\sigma_{{\text{v}}} = \gamma H,\;\sigma_{{\text{H}}} = \lambda \gamma H\;{\text{and}}\;\lambda = \frac{\mu }{1 - \mu }$$

where $\sigma_{v}$ is the vertical stress (MPa), $\sigma_{H}$ is the horizontal stress (MPa), *H* is the depth (m), $\mu$ is the Poisson’s ratio, and $\lambda$ is the lateral pressure coefficient.

The Poisson’s ratio of a rock is basically independent of the type of rock but is related to its apparent strength, weathering degree, and the degree of jointing and cracking. Generally, it is reasonable to consider the following rock masses: grade V, with values of 0.3–0.35; grade IV, with values of 0.3–0.25; grade III, with values of 0.25–0.2; or grade II, which is high-strength granite, with a value of 0.2. If the rock is broken, its Poisson’s ratio is quite high. When the Poisson’s ratio of the rock is in the range of 0.2–0.35, the lateral pressure coefficients are 0.25–0.54. In fact, the Poisson’s ratio of rocks in rockburst mines rarely exceeds 0.3. Thus, the critical value is further lowered, and the corresponding lateral pressure coefficient is 0.43.

When the actual measured horizontal stress exceeds the critical value ($\lambda \gamma H$) (Eq. 2), the stress field is considered a tectonic stress field.

2 $$\sigma_{{{\text{HM}}}} > \sigma_{{\text{H}}} = \lambda \gamma H$$

where ${\sigma }_{HM}$ is the actual measured horizontal stress (MPa).

### Chinese stress field and rockburst classification

A famous Chinese geologist, Siguang Li, pointed out that “under the condition that the effect of tectonic stress only affects a certain thickness of the upper crust, the importance of the horizontal stress component far exceeds the vertical one”^32^. In the 1950s, Hast found that the maximum principal stress of the upper crust was almost horizontal or close to horizontal when measuring the in situ stress, and the maximum horizontal principal stress was generally 1–2 times the vertical stress^33^. This fundamentally subverted the viewpoint that in situ stress is hydrostatic stress and vertical stress is the principal stress.

The stress field of the current crust in China, particularly tectonically active areas, is dominated by horizontal stress. Statistical analysis of in situ stress measurements, macroscopic seismic surveys, microscopic displacement measurements of faults, and focal mechanism solutions indicate that the current in situ stress field in China, particularly tectonically active areas, is dominated by horizontal stress, and the horizontal component of in situ stress is approximately 1.5 times the vertical stress component. The shallow stress field is nonuniform, and the stress orientation and relative strength vary based on location. The measured stress fields in some areas in China are shown in Table 1^34^, and the measured data based on the geo-dynamic division in Chinese coal mines are shown in Table 2. The ratio of the maximum horizontal principal stress to the vertical principal stress in most Chinese coal mines ranges from 0.5 and 2.5, which indicates that in most cases, the maximum horizontal principal stress is higher than the vertical stress. Both the statistical and measured data do not obey the Zernike hypothesis. Most coal mines are in the tectonic stress field, and coal mine rockbursts are tectonic stress-driven.

**Table 1:** Ratios of the measured maximum horizontal principal stress to the vertical stress in China.

Distribution	< 1	1–2	2–3	3–4	> 4
Measurement point	231	779	117	9	2
Ratio	20.3%	68.4%	10.3%	0.8%	0.2%

**Table 2:** Relationship between the measured maximum horizontal principal stress and vertical stress components in some Chinese coal mines.

Coal mine	Depth (m)	Maximum principal horizontal stress	Vertical stress/horizontal component	Principal horizontal stress/Vertical stress	Stress field type	Poisson’s ratio/lateral pressure coefficient
Wudong coal mine	275	7.0	7.4/2.1	0.95	Tectonic stress field	0.22/0.28
Jixian coal mine	668	36.61	18.0/7.8	2.03	Tectonic stress field	0.30/0.43
Laohutai coal mine	758	31.94	20.5/8.4	1.56	Tectonic stress field	0.29/0.41
Yuejin coal mine	996	22.87	26.9/11.0	0.85	Tectonic stress field	0.29/0.41
Linxi coal mine	980	46.27	26.46/7.4	1.75	Tectonic stress field	0.22/0.28

According to section “Rockburst classification”, based on the classification method of rockbursts in the coal and rock mass stress state, the above analytical results show that most coal mines in China are in the tectonic stress field. Therefore, the rockburst type in coal mines is the tectonic stress type. Coalfields in China experience dynamic disasters, such as rockbursts, and the stress field is mainly tectonic stress. In the shallow region, the tectonic stress is obvious. After progressively deepening, the stress field gradually transitions into a situation where gravity–tectonic stress is roughly equivalent.

### Direction of the modern Chinese tectonic stress field

**Figure 3:**
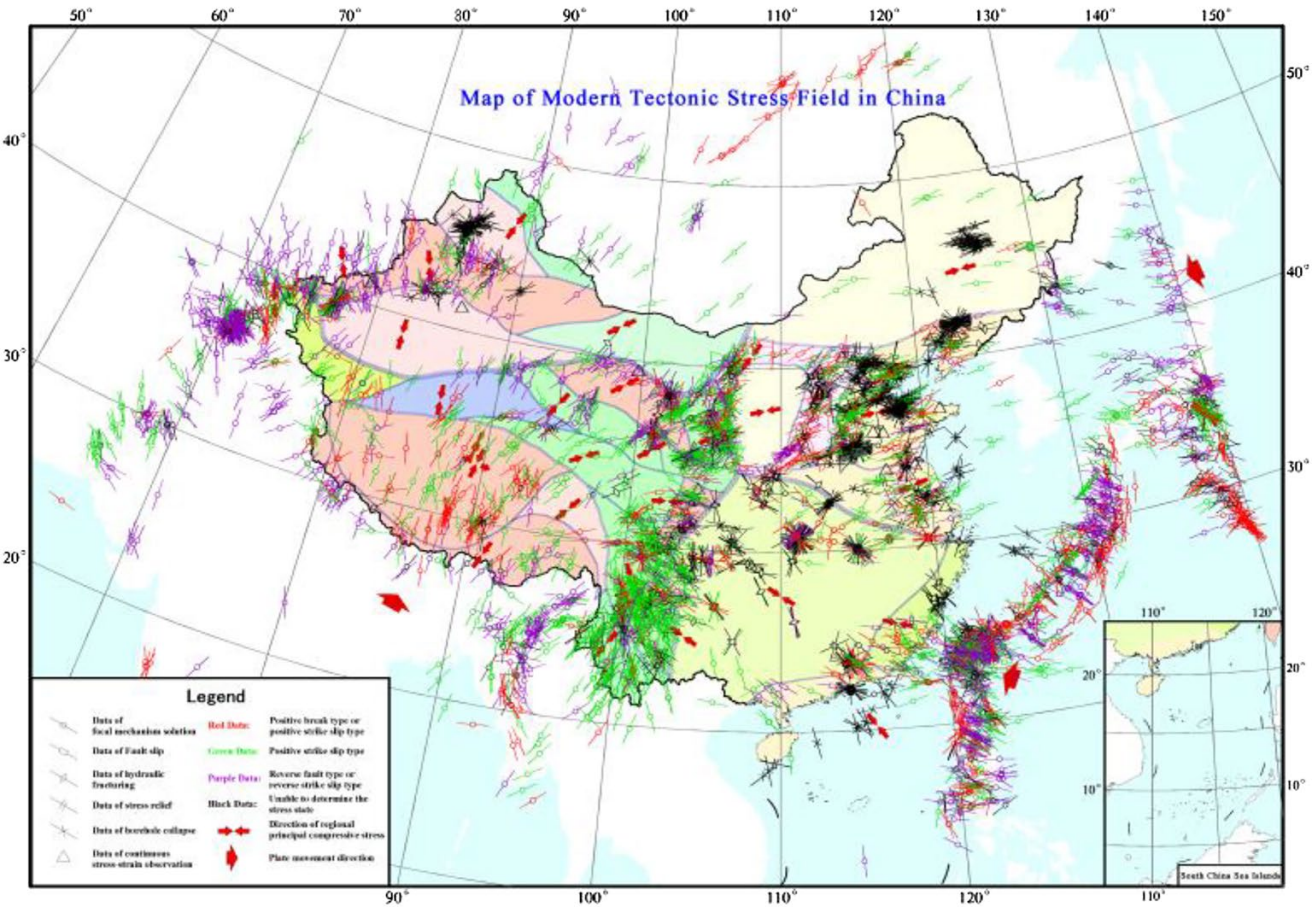
Tectonic stress field map of China^38–40^**.**

Studies (Zoback and Zoback^35^) have shown that in a large area, the direction of tectonic stress is relatively stable, follows a certain distribution, and has a certain relationship with the geological structure and modern crustal movement. The in situ stress distribution in China is zoned; that is, not only are the directions very different but also the values are different. In eastern China and adjacent areas, the modern tectonic stress field is mainly oriented in the WNW–EW direction^36,37^, and this orientation mainly features the characteristics of the current tectonic activity in the new Chinese system. The maximum principal compressive stress direction in the west is mostly nearly SN. The in situ stress magnitude is commonly higher than the national average value, including in the NE and NW directions, and this magnitude mainly exhibits the characteristics of the current tectonic activity in the western regions, Hexi system, and anti-“S” type stress (Fig. 3)^38–40^.

Among them, the main compressive stress direction in North‒Northeast China is ENE–WSW, while dominant SE‒NW to ESE–WNW characteristics in South China are very obvious. In western China and adjacent areas, the main compressive stress direction is NNE‒NNW. At the northern and eastern edges of the Qinghai–Tibet Plateau, the main compressive stress direction changes from NNE‒SSW to NNE‒NNW^41^. In the southern part of the Qinghai–Tibet Plateau, the main compressive stress direction is nearly E‒W; around the Ordos plate, the modern tectonic stress is mainly tensile, and the stress direction and structure are obviously different from the tectonic stress field in North China^42^. In the East Pacific, adjacent to eastern China, the principal compressive stress direction of the modern tectonic stress field is nearly E‒W, and the stress structure is both reverse fault and strike-slip^43^. In the Afghanistan and Pamir regions, adjacent to western China, the main compressive stress direction of the modern tectonic stress field is nearly S–N, and the stress structure is dominated by extrusion. The Altai to Baikal area adjacent to northern China is the main modern compressive tectonic stress field. The direction of compressive stress is NNE‒SSW.

A large amount of seismic data and measured data of in situ stress indicate that the maximum and minimum principal stress axes of the modern tectonic stress field in China are both horizontal. The direction of the Chinese tectonic stress field determined based on modern tectonic movement can guide in situ stress measurements. For a coal mine that has in situ stress measurements, when the measured in situ stress direction is basically consistent with the direction of the modern tectonic stress field, the measured stress field direction can represent the stress direction of the external geological mass in the coalfield. If a large discrepancy in the direction is present, it can represent only the direction of the stress field at the location of the measurements.

### Importance of the influence of tectonic stress on rockbursts

A high-stress area, stress-gradient area and low-stress area naturally form within the scope of the coal mine field due to the interaction between structural fault blocks and the difference in rock mechanical properties. In the high-stress area, under the action of high stress, the accumulated elastic energy of the rock is much higher than that of the normal-stress area. Some rock masses have reached the critical point of steady-state to unsteady-state transition, which is most likely to lead to rockbursts. In the stress-gradient area, the stress and deformation modulus of rocks increases greatly, the brittleness of rocks increases, but the failure strength decreases; thus, the geological structure easily forms, and rockbursts are easily induced under the action of tectonic stress. In low-stress areas, the rock properties have little change and does not easily generate energy accumulation, with the lowest risk of rockburst^29,44^.

Ren^45^ studied the evolutionary pattern of rockbursts in roadways with deep high tectonic stress by means of experimental and theoretical research. He believed that the horizontal tectonic stress played an important role in the development of rockbursts. When the horizontal tectonic stress is small, the cracks in coal and rocks expand stably only under the action of the compressive load, and no rockburst occurs. Under the action of high-level stress, the coal and rock interior bear the tensile load, the crack expands unstably, the accumulated energy releases instantaneously, and rockbursts occur. When the horizontal tectonic stress continues to increase, the system composed of surrounding rock and coal slides elastically overall, and the energy is jointly released by the broken coal and surrounding rock, which leads to a higher-strength rockburst.

Song^46^ researched tectonic stress and its influence on rockbursts in the Tangshan mining area, China. He believed that tectonic stress had an obvious influence on the distribution of the stress field and energy field in the coalbed and its surrounding rock. Under high tectonic stress conditions, high horizontal stress is the source of force and elastic strain energy stored in the coalbed, and its surrounding rock is the source of energy to induce rockbursts during coal mining.

Han^47^ analyzed the inner relationship between the in situ stress field and coal and gas outbursts, rockbursts, water inrush and other dynamic coal and rock disasters. According to the research results, the in situ stress field in the Kailuan mining area is part of the Earth dynamical field, the horizontal tectonic stress is dominant, and it is part of the high-stress zone. The in situ stress magnitude and orientation are controlled by the Kaiping syncline. The stress value is the highest in the Kaiping synclinal axis section. Far from the axis, the stress value decreases gradually. The maximum principal stress orientation and axis trend of the Kaiping syncline are approximately vertical. In the Kailuan mining area, coal and gas outbursts and rockbursts occurred in the synclinal axis section of Kaiping, where the stress value is the highest, and water inrush occurred in the Kaiping syncline wing, where the stress value is the lowest. The dynamic coal and rock disaster in the Kailuan mining area occurred in a unified tectonic stress environment.

The importance of the influence of tectonic stress on rockbursts and other disasters in coal mines and other mines has also been described in other papers^48–54^.

## Geo-dynamic environment and dynamic rockburst mechanism

### Modern tectonic movement and geo-dynamic environment

Since rockbursts in coal mines are tectonic stress-driven, we first need to study modern tectonic movement and the modern tectonic stress field. From the perspective of modern tectonic movement, 50 million years ago, the Indian plate collided with the Eurasian continent, and the Himalayas formed, creating the tectonic pattern of the Chinese mainland. The geo-dynamic environment of the coal mine has been inevitably affected and controlled by this tectonic pattern and is closely related to modern tectonic movement. The stress changes and energy transfer caused by tectonic movement affect the engineering geological media of the coal mine. The geo-dynamic environment is an overall evaluation of the tectonic, movement and stress characteristics of the regional geological media where the coal mine is located. We aim to reveal the dynamic effects of tectonic forms, tectonic movements, tectonic stress, strata characteristics, and their combination on coal and rock masses in coal mines under natural geological conditions. The geo-dynamic environmental evaluation method can be used to determine how coal mine (mining area) geo-dynamic evolution and modern tectonic movement affect coal mine rockburst occurrence^27^.

Modern tectonic movement redistributes stress and energy in tectonic plates (coal and rock masses) in the crust to achieve a new stress balance. The results of tectonic movement can be divided into the following three areas according to the theory of geo-dynamic division^24^:

1. areas where stress and energy are relatively stable;
2. areas where stress and energy increase (maximum at the critical state);
3. areas where stress and energy exceed the strength limit of rock.

Coal mining in the first area causes only conventional mine pressure, and no dynamic coal mine disasters, such as rockbursts, occur. The crustal rock in the third area is in a state of instability and is released by earthquakes, and this area reaches a new energy balance and changes into the first and the second areas. With coal mining in the second area, under the tectonic stress field, the coal and rock mass stress and energy are in dynamic balance, and energy accumulates locally in the coal and rock mass. Under the disturbance of engineering activities, such as coal mining, the energy increases, leading to system instability, energy release, and dynamic coal mine disasters, such as rockbursts. How to further divide the risk area of rockbursts in the mine field is an important research topic for rockbursts.

According to the geo-dynamic division theory, dynamic coal mine disasters, such as rockbursts, should occur in a suitable geo-dynamic environment, and rockbursts are the result of the combined effect of the geo-dynamic environment and mining disturbance. To study rockbursts in coal mines, we should first examine the modern tectonic stress field and coal mine geo-dynamic environment, determine the factors influencing the geo-dynamic environment, and reveal the nucleation, evolution and development conditions of rockbursts in the engineering geological mass of the coal mine. We also need to elucidate the effect of the dynamic background of the geological mass on rockbursts, quantify the grade of the geo-dynamic environment, and determine the type of geo-dynamic environment in coal mines.

### Geo-dynamic environment and rockbursts

According to the evaluation of the geo-dynamic environment, coal mines can be classified into typical rockburst coal mines, atypical rockburst coal mines, and non-rockburst coal mines. For a typical rockburst coal mine, the dynamic environment is dominant. When the energy provided by the geo-dynamic environment exceeds the critical energy required for a rockburst, a rockburst occurs. When the energy provided by the geo-dynamic environment is insufficient to exceed the critical energy of a rockburst, mining disturbance supplies extra energy, and thus, the total energy is higher than the critical energy, and an atypical rockburst occurs. If the total energy cannot surpass the critical energy, the coal mine is a non-rockburst type coal mine.

**Figure 4:**
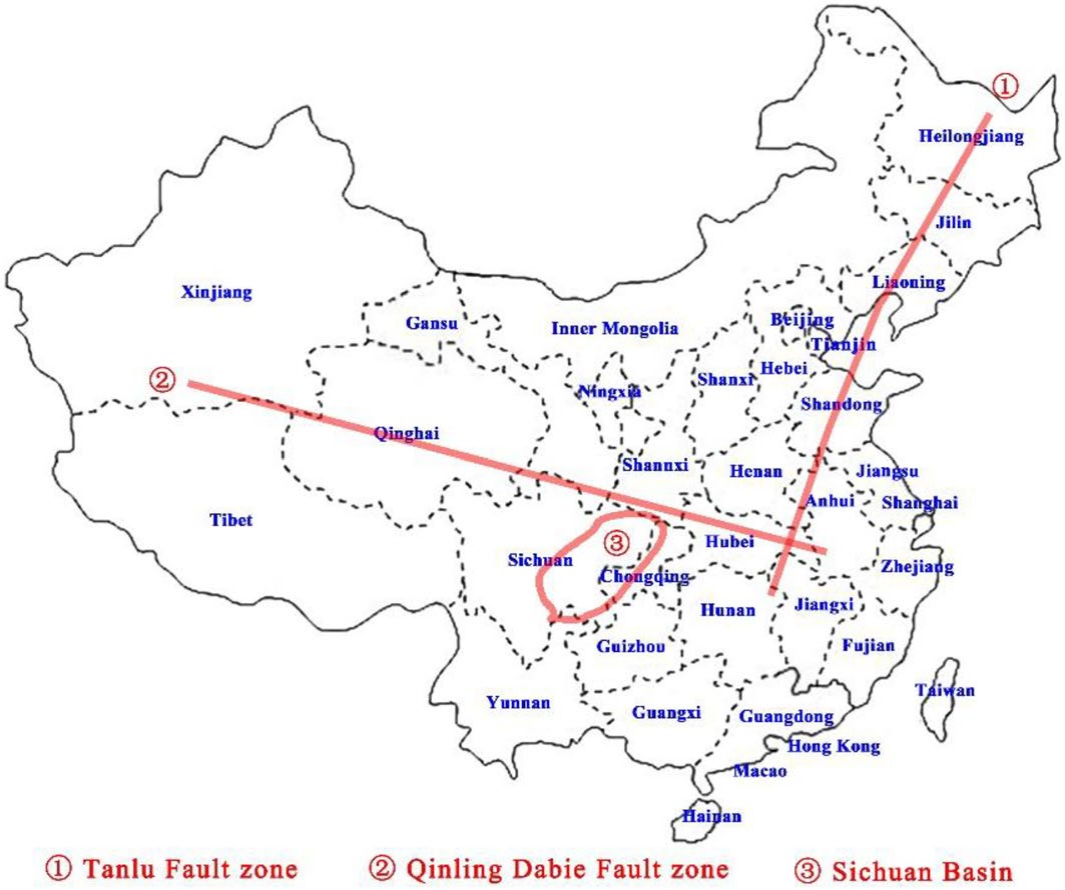
“110” zoning of dynamic coal mine disasters based on the modern tectonic system.

Extensive studies on the distribution of dynamic coal mine disasters, such as rockbursts and coal and gas outbursts, show that the occurrence of dynamic coal mine disasters is nonuniformly distributed in time and space and displays a regional distribution. The nonuniform spatial distribution of dynamic coal mine disasters mainly depends on the type and characteristics of modern tectonic plates. The nonuniform temporal distribution mainly depends on the active time, pattern of modern tectonic plates and tectonic stress distribution in coal and rock masses. It is also related to solar activity, solid tides, and the irregularity of the Earth’s rotation speed in a certain period and season. According to the distribution characteristics of the modern Chinese tectonic system, the distribution of dynamic coal mine disaster areas can be preliminarily called the “110” distribution (Fig. 4), which includes the Tanlu fault zone, encompassing Hegang, Shuangyashan, Qitaihe, Jixi Liaoyuan, Fushun, Shenyang, Kailuan, Yanzhou, Xinwen, Zaozhuang, Xuzhou, Huaibei, and Huainan; the Qinling Dabieshan fault zone, including Huating, Yima, Pingdingshan, Zhengzhou, Jiaozuo, and Huainan; and the Sichuan Basin, containing Tianfu, Songzao, Nantong, Huaying Mountain, and Zhongliang Mountain. On this basis, other modern tectonic systems affecting and controlling dynamic coal mine disasters can be further divided.

### Dynamic mechanism of rockbursts

The current viewpoint is that the burst proneness, rock structure, geological structure, mining depth, periodic pressure, hard and thick roof, support pressure, coal pillar stress, stress concentration, goaf “square” pressure, and advancing speed are the main factors^55^. Coal mines with rockbursts must meet all or some of the above conditions, indicating that they are indeed the main factors influencing rockbursts. However, coal mines without rockbursts also meet some of the above conditions, but rockbursts do not occur. This shows that in addition to the above influential factors, other factors restricting the occurrence of rockbursts exist. It is necessary to clarify the source of the “accumulated elastic energy” provided by other controlling factors. First, coal mining is not the entire source. Otherwise, more than 5000 coal mines in China should have dynamic coal mine disasters, such as rockbursts. Since not all are accumulated by coal mining, what about the other channels for accumulation?

Coal mine dynamic disasters are caused by the energy in the coal and rock masses in the engineering area. The dynamic basis is energy accumulation. Therefore, determination of the conditions for energy accumulation is a prerequisite to rockburst research. Geo-dynamic division theory shows that energy accumulation mainly comes from two aspects, namely, natural geological conditions and mining engineering conditions. Energy accumulation in coal and rocks under natural conditions is closely related to the tectonic stress field, which is caused by modern tectonic movement. There are two kinds of natural geological conditions. One is the geo-dynamic environment that can produce dynamic disasters in coal mines, and the other is that the geo-dynamic environment is absent. Therefore, the study of the geo-dynamic environment can inform in advance whether the geological mass in the area where mining engineering is located has a geo-dynamic environment for dynamic coal mine disasters; this information is based on evaluation indicators, such as modern tectonic movement and modern tectonic stress fields, which can explain why certain mining areas never occur. In contrast, other mining areas have frequent dynamic coal mine disasters. The core concept of applying geo-dynamic division methods to predict dynamic coal mine disasters is based on the following understanding. First, dynamic coal mine disasters must have corresponding geological energy. The environment is affected by many factors. Second, dynamic coal mine disasters possess different modes under different mining areas, different coal mines, different coal seams, and different tectonic and stress conditions^23^. Third, the accurate prediction of the time and location of the occurrence of the disasters is extremely difficult, but it is possible to predict the probability (probability of occurrence) of the disasters.

Energy accumulation is the foundation of rockbursts. Petukhov^56^ believes that the entire system of “rocks surrounding the coal seam” should be involved in the formation and manifestation of dynamic coal mine disasters. From the energy viewpoint, each stress and strain state of the rock corresponds to an energy state. The dynamic coal and rock system always exchanges energy with the exterior, stores the energy transferred from the exterior, and releases energy to the exterior in some form to maintain energy balance. When the system is unstable, it appears as a rockburst. The dynamic mechanism of dynamic coal mine disasters is the existence of energy in the coal and rock mass in the engineering area. Its dynamic base is energy accumulation and the dynamic mechanism of rockbursts, i.e., the energy source provided by the external geological mass and the engineering activities.

### “Three condition” criteria of rockbursts

The viewpoint of geo-dynamic division states that the energy source of rockbursts must include the external geological mass around the coalfield. Coal mine dynamic disasters, such as rockbursts, are the result of the coupled effect of the geo-dynamic environment and mining disturbance. Under the conditions of the coal seam (or its roof and floor strata) having burst proneness and the burst evaluation being a high-impact risk, “three conditions” for rockburst occurrence are proposed.

A geo-dynamic environment is a necessary condition for the occurrence of dynamic disasters, such as rockbursts, in coal mines. When there is no geo-dynamic environment conducive to rockbursts in a mining area, a rockburst does not occur. When a geo-dynamic environment exists, only the conditions for rockbursts exists. Therefore, an analysis of the geo-dynamic environment serves as the basis for predicting the risk of dynamic disasters, such as rockbursts, in coal mines.

Mining disturbance is a sufficient condition for the occurrence of rockbursts. The sufficient condition is negative if the coal mine is not mined, and no rockburst occurs. An unreasonable mining layout and settings further increase the risk and eventually induce a rockburst. The risk-releasing measure is a controlling condition for rockburst mitigation, and the controlling condition affirms the "conclusion"; that is, when this condition is not met (mining without implementing measures to relieve danger), a rockburst will certainly occur. When this condition is met, the mine that has implemented measures to relieve danger, including prevention techniques and protective measures, contains prevention and control technology. By adopting reasonable and effective prevention and control measures, prevention and treatment effects can be achieved, risk can be eliminated, and safe production can be realized. The criterion can reasonably explain why most Chinese mining areas predict that rockbursts and other dynamic coal mine disasters are relatively large and dangerous, whereas the phenomenon of dynamic disasters in actual mine production is comparatively much less apparent.

## Conclusions

1. Dynamic coal mine dynamic disasters, such as rockbursts, are based on modern tectonic movement and modern tectonic stress fields. The stress change and energy transfer caused by tectonic movement inevitably affect the engineering geological media and coalfield where the coal mine is located. Without a full-scale consideration, simple action is impracticable.
2. A method to discriminate the tectonic stress field is proposed. Actual measurements show that most coal mines in China have only tectonic stress fields. In the current mining depth range, rockbursts should be tectonic stress-driven. The direction of the Chinese tectonic stress field determined based on modern tectonic movement guides in situ stress measurements.
3. Dynamic coal mine disasters, such as rockbursts, are evidence of stored energy in the coal and rock mass in the mining area. Geo-dynamic division theory believes that the conditions for energy accumulation are mainly natural geological dynamic conditions and mining engineering conditions, and the driving force of rockbursts is the result of the combined effect of energy provided by geological bodies and energy provided by engineering activities.
4. Dynamic coal mine disasters, such as rockbursts, must reside in a suitable geo-dynamic environment, which is affected by many factors. The geo-dynamic environmental evaluation method can be used to determine the degree of influence of the coal mine (mining area) on the geo-dynamic evolution process and modern tectonic movement on the mine field. According to the distribution of the modern Chinese tectonic system, dynamic coal mine disasters, such as rockbursts, follow a “110” distribution.
5. According to the geo-dynamic environmental evaluation method, the rockburst classification is determined. A “three condition” criteria of rockburst is proposed, namely, the geo-dynamic environment is a necessary condition for rockbursts, mining disturbance is a sufficient condition for rockbursts, and risk-releasing measures are a controlling condition for rockburst risk mitigation.

## Supplementary Information

Supplementary Information.

## Data Availability

All data generated or analysed during this study are included in this published article and its supplementary information files.
