# Supplementary material for: Insights into fundamental problems of rockburst under the modern structure stress field
Source: Sci Rep. 2022 Nov 24;12:20299. doi: 10.1038/s41598-022-24857-4 (PMC9700775; doi:10.1038/s41598-022-24857-4)
Supplement: Supplementary file 1 — Supplementary Information. [file 41598_2022_24857_MOESM1_ESM.docx]

Title：

Insights into the fundamental problems of rockbursts under the modern structural stress field

Author list：

Hai Rong Email: ronghai1988@163.com

Nannan Li Email: 934744832@qq.com

Hongwei Zhang Email: 815367982@qq.com

Dequan Sun Email: [1452847301@qq.com](mailto:1452847301@qq.com)

Bingjie Huo Email: [huobingjie@163.com](mailto:huobingjie@163.com)

Data for revised figure 1

The number of rockburst coal mines in China

| Year | the number of rockburst coal mines in China | the total number of  coal mines in China | the proportion of  rockburst coal mines |
| --- | --- | --- | --- |
| 1985 | 32 | 80000 | 0.0004 |
| 1990 | 53 | no data | no data |
| 2000 | 70 | 30000 | 0.002333 |
| 2010 | 93 | 23000 | 0.004043 |
| 2012 | 142 | 18000 | 0.007889 |
| 2015 | 177 | 10800 | 0.0148 |
